# The visual magnocellular deficit in Chinese-speaking children with developmental dyslexia

**DOI:** 10.3389/fpsyg.2014.00692

**Published:** 2014-07-03

**Authors:** Yi Qian, Hong-Yan Bi

**Affiliations:** ^1^Key Laboratory of Behavioral Science, Institute of Psychology, Chinese Academy of SciencesBeijing, China; ^2^University of Chinese Academy of SciencesBeijing, China

**Keywords:** developmental dyslexia, magnocellular pathway, coherent motion detection, orthographic processing skills, Chinese reading

## Abstract

Many alphabetic studies have evidenced that individuals with developmental dyslexia (DD) have deficits in visual magnocellular (M) pathway. However, there are few studies to investigate the M function of Chinese DD. Chinese is a logographic language, and Chinese characters are complicated in structure. Visual skills and orthographic processing abilities are particularly important for efficient reading in Chinese as compared to alphabetic languages. Therefore, it is necessary to investigate the visual M function of Chinese DD and whether the M function was associated with orthographic skills. In the present study, 26 dyslexic children (mean age: 10.03 years) and 27 age-matched normal children (mean age: 10.37 years) took part in a coherent motion (CM) detection task and an orthographic awareness test. The results showed that dyslexic children had a significantly higher threshold than age-matched children in CM detection task. Meanwhile, children with DD responded more slowly in orthographic awareness test, although the group difference was marginally significant. The results suggested that Chinese dyslexics had deficits both in visual M pathway processing and orthographic processing. In order to investigate the relationship between M function and orthographic skills, we made a correlation analysis between CM threshold and orthographic awareness by merging performance of dyslexic children and age-matched children. The results revealed that CM thresholds were positively correlated with reaction times in orthographic awareness test, suggesting that better M function was related to better orthographic processing skills.

## INTRODUCTION

Developmental dyslexia (DD) is a neurobiological reading disorder. Individuals with DD have difficulties in accurate or fluent word recognition, spelling, and word decoding despite adequate instruction and intelligence (Lyon et al., 2003). Although it is widely accepted that there are phonological deficits in DD, some researchers indicate that dyslexia can be traced back to a more general perceptual dysfunction. Magnocellular (M) deficit theory postulates that the core deficit of DD is the impairment in M pathway, which is specialized for temporal processing (Stein and Walsh, 1997; Stein, 2001).

In alphabetic languages, phonological information of words can be activated according to grapheme–phoneme correspondence (GPC) rules. Efficient auditory function is essential for phonological processing (Boets et al., 2006). Tallal and Piercy (1973) and Tallal (1980) first found individuals with dyslexia performed worse than typical readers in discriminating rapid speech and non-speech stimuli. Later, many studies consistently found that dyslexics showed poor performance on a number of auditory tasks, including frequency discrimination (McAnally and Stein, 1996; Ahissar et al., 2000) and temporal order judgment (Nagarajan et al., 1999; Schulte-Körne et al., 1999). Longer intersound intervals were needed for dyslexics to perceive an illusory auditory saltation or follow each successive sound in a continuous fashion, suggesting a prolonged “cognitive integration window” (Hari and Kiesila, 1996; Helenius et al., 1999). The deficits in temporal auditory processing were also confirmed in event-related potential (ERP) and functional magnetic resonance imaging (fMRI) studies (e.g., McAnally and Stein, 1997; Kujala et al., 2000, 2003; Temple et al., 2000; Paul et al., 2006; Stoodley et al., 2006; Gaab et al., 2007; Khan et al., 2011). These results consistently revealed that dyslexics have deficits in temporal auditory processing in alphabetic languages.

With respect to visual processing skills, dyslexics also exhibit deficits in visual M pathway. Vidyasagar and Pammer (2010) indicated that reading can be affected by a deficit at any step along the visual M pathway, which stretches from the retina to the posterior parietal cortex, including middle temporal area (MT/V5; Boden and Giaschi, 2007). Many studies found that dyslexics were less sensitive to coherent motion (CM) than age-matched controls (Cornelissen et al., 1995; Talcott et al., 1998, 2003; Witton et al., 1998; Hansen et al., 2001; Conlon et al., 2004; Pellicano and Gibson, 2008), reflecting the deficient M processing of DD. Pre-reading children at familial risk for DD exhibited the disability in detecting CM, suggesting deficits in M pathway occur before reading commencement (Kevan and Pammer, 2008). The deficient CM detection was persistent and not affected by stimulus duration, dot density or practice (Talcott et al., 2000a; Conlon et al., 2009; Wright and Conlon, 2009). Slaghuis and Ryan (2006) found CM sensitivity in mixed subgroup of dyslexics was significantly lower than that in normal group, but CM sensitivity in surface and phonological DDs was not different from that in normal readers. In a meta-analysis study, larger effect sizes were obtained for adult subjects compared with children, suggesting CM deficit was more reliable in dyslexic adults (Benassi et al., 2010). However, some studies didn’t support M theory of dyslexia. Ramus et al. (2003) found that only 2 of 16 dyslexic adults had visual M deficit. The low incidence, together with that the two visually impaired dyslexics also had auditory and phonological problems, might not confirm that visual M deficit was an independent core deficit of DD. Sperling et al. (2005) pointed out that deficits in noise exclusion, not M processing, contributed to the etiology of dyslexia. In the high-noise conditions, dyslexic children’s contrast thresholds were significantly higher than non-dyslexic children’s in both M and parvocellular (P) pathways. But in the no-noise conditions, contrast thresholds of dyslexic and non-dyslexic children did not significantly differ in either M or P pathway. The results suggested that dyslexics had deficits in noise exclusion rather than M processing. However, Conlon et al. (2012) discussed that DD’s difficulty in noise exclusion was the consequence of a sensory processing deficit in the M or dorsal stream. One explanation of noise exclusion was greater internal noise in the visual system, which was evidenced by the small number and disorganized manner of neurons in the M and dorsal stream. In addition, dyslexics had normal coherent form thresholds (Conlon et al., 2009), which could not be interpreted by noise exclusion theory. Skottun (2011) indicated that area MT receives inputs from M pathway as well as P and koniocellular pathways. CM sensitivity could not be only attributed to M pathway. As a result, he claimed CM detection might not be a reliable test of M processing. Nevertheless, he also underlined that the results should not be taken to mean that M deficiencies have no effect on motion perception or M deficits do not have the potential to create deficient motion perception. In fact, CM sensitivity was still a widely accepted test to measure M processing, although there were a lot of questions to be answered. Apart from the above problems, there was another question: was CM deficit general for different languages? A study found that poor readers in Thai were less sensitive to detect CM, while poor readers in Korean were not. It might result from the fact that Korean script was more complex than Thai. The authors thought the visual complexity of a script might modulate the expression of M pathway deficits in DDs (Kim et al., 2004).

Chinese is a logographic language without GPC rules. Chinese character is visually compact (Ho et al., 2004) and looks like a two-dimension picture (Zhang et al., 2006). Visual skills are particularly important for Chinese reading (Chung et al., 2008; Li et al., 2012; Yang et al., 2013). Additionally, Chinese dyslexic children have deficits in multiple cognitive skills, including phonological awareness, morphological awareness, rapid naming and orthographic awareness (Huang and Hanley, 1995; Ho and Lai, 1999; Ho et al., 2002, 2004; Shu et al., 2006). Thereinto, orthographic processing deficit is one of the most dominant defects in Chinese DD (Ho et al., 2004). As known, orthographic processing needs efforts in visual analysis. Then, are orthographic processing skills associated with M function? Previous alphabetic studies revealed CM sensitivity was related to orthographic processing skills (Talcott et al., 2000b). Skilled readers who excelled at motion detection performed better in a lexical decision task than those who are poor at detecting CM (Levy et al., 2010). In Chinese character reading, visual analysis and orthographic processing were specifically required. A prior study found Chinese children with dyslexia showed reduced amplitude of visual mismatch negativities (vMMNs) than both age-matched and reading level matched children in the visual M condition, whereas there was no difference in auditory mismatch negativities (aMMNs) of auditory modality between dyslexic children and the two control groups. This result suggested Chinese dyslexic children only had deficits in visual M pathway, while the auditory temporal processing skills were intact (Wang et al., 2010). Meng et al. (2011) found Chinese dyslexics had significantly higher CM threshold than age-matched children, which also confirmed the visual M pathway impairment in Chinese DD. Additionally, Meng et al. (2011) revealed the CM threshold made a significant contribution to the speed of orthographic similarity judgment in a random sample. However, orthographic similarity judgment might be not a proper task to measure orthographic awareness, because the stimuli were all real characters. There were no non-characters violating orthographic rules. Processing in this task only involved simple form comparison. It was unnecessary for children to judge whether a character conformed to orthographic rules or not, which reflected orthographic processing. In the present study, we adopted a lexical decision task, in which children were required to judge whether the target character was a real character. There were three kinds of characters: real character, pseudo-character (orthographic-legal) and non-character (orthographic-illegal). By comparing the group difference in rejecting pseudo-characters and non-characters, we investigate orthographic awareness deficits in Chinese dyslexic children.

Therefore, there are two aims in the current study. The first aim is to investigate the deficits of Chinese dyslexics in visual M pathway and orthographic awareness. The second aim is to explore the relationship between visual M function and orthographic processing ability.

## MATERIALS AND METHODS

### PARTICIPANTS

Twenty-six dyslexic children [6 females and 20 males, mean age: 10.03 years (range: 9–11 years)] and twenty-seven age-matched controls [CA, 9 females and 18 males, mean age: 10.37 years (range; 9–11 years)] took part in the study. The children were recruited from ordinary primary schools in Beijing. The study was conducted under the informed consent of parents, and was approved by the Institutional Review Board of the Institute of Psychology, Chinese Academy of Sciences. All of the participants were right-handed, and had normal hearing and normal or corrected-to-normal vision without ophthalmological or neurological abnormalities. The inclusionary criteria for dyslexics were that the IQ was above 85 as measured by Raven’s Standard Progressive Matrices (Raven et al., 1996), while the written vocabulary test score was at least one and a half standard deviations below corresponding age norm in the Standard Character Recognition Test (Wang and Tao, 1996). This was a widely used test for screening Mandarin-speaking Chinese dyslexia children in Mainland China (e.g., Shu et al., 2006; Li et al., 2009; Wang et al., 2010; Meng et al., 2011). In this test, children were asked to write down a compound word based on a character (constituent morpheme) provided on the sheet. The characters were selected based on the grades. The performance was measured by adding the total number of correct characters the participants could make use of in word-composition and the constant which was the number of characters almost all children in this grade could recognize. Additionally, rapid naming speed was tested. Digits (2, 4, 6, 7, and 9) were repeatedly presented visually in random order on a six row × five column grid. Children were asked to name each digit in sequence as quickly as possible. The total time (s) was collected. Characteristics of participants were shown in **Table 1**.

**Table 1:** Characteristic of dyslexics and age-matched controls.

	DD (*n* = 26)	CA (*n* = 27)	*p*
Age (years)	10.03 (0.46)	10.37 (0.90)	>0.05
IQ	110.27 (12.99)	114.22 (9.92)	>0.05
Vocabulary	1113.16 (327.81)	1864.61 (324.87)	<0.001
Time of rapid naming (s)	13.31 (2.76)	10.82 (2.29)	=0.001

### ORTHOGRAPHIC AWARENESS TEST

This task was consisted of 40 real characters, 20 pseudo-characters, and 20 non-characters. Pseudo-characters (*e.g.*, 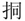) were made up of two position-legal radicals. The radicals of non-characters (*e.g.*, 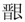) were in illegal positions. Pseudo-characters conformed to orthographic rules, while non-characters did not. A lexical decision task was adopted, participants were asked to judge whether a presented item was a real character. So, the correct response to a real character was “yes,” but to a pseudo-character or a non-character was “no.” The task was presented in a computer, which after a 500-ms fixation, each character was presented in isolation in the center of the computer screen until participants responded (the longest duration was 3000 ms). Although pseudo-characters and non-characters are not real characters, pseudo-characters conformed to orthographic rules while non-characters didn’t. So, the different performance between pseudo-character and non-character judgment reflected the orthographic skills. Therefore, only reaction time (RT) and accuracy in pseudo-character and non-character responding were recorded. The reliability (Cronbach’s Alpha) was 0.804.

### COHERENT MOTION DETECTION

The CM task was similar to that in the study of Solan et al. (2004). Two patches of 300 randomly moving white dots with a speed of 7^∘^/s and a lifetime of 225 ms were presented on the left and right sides of screen with dark background. The luminance of dots was 125 cd/m^2^, and the luminance of background was 0.39 cd/m^2^, Michelson contrast was 99.4%. The patches were 10^∘^ wide and 14^∘^ high, separated by 5^∘^, and were presented for 2300 ms in each trial. In one patch, all dots moved randomly, while the other patch had a certain percentage of dots moving coherently leftward and rightward. Participants had to judge which patch had such coherently moving dots after patches disappeared. CM threshold was varied according to a 1-up-1-down staircase procedure. Incorrect responses led to an increase in the number of coherent moving dots by 1%, while correct responses led to a decrease by 1%. After 10 reversals, a session was terminated. Threshold was defined by the mean of the number of coherent moving dots of the last six reversals. The experiment included three sessions, and the thresholds were averaged as the final CM threshold presented here.

## RESULTS

**FIGURE 1:**
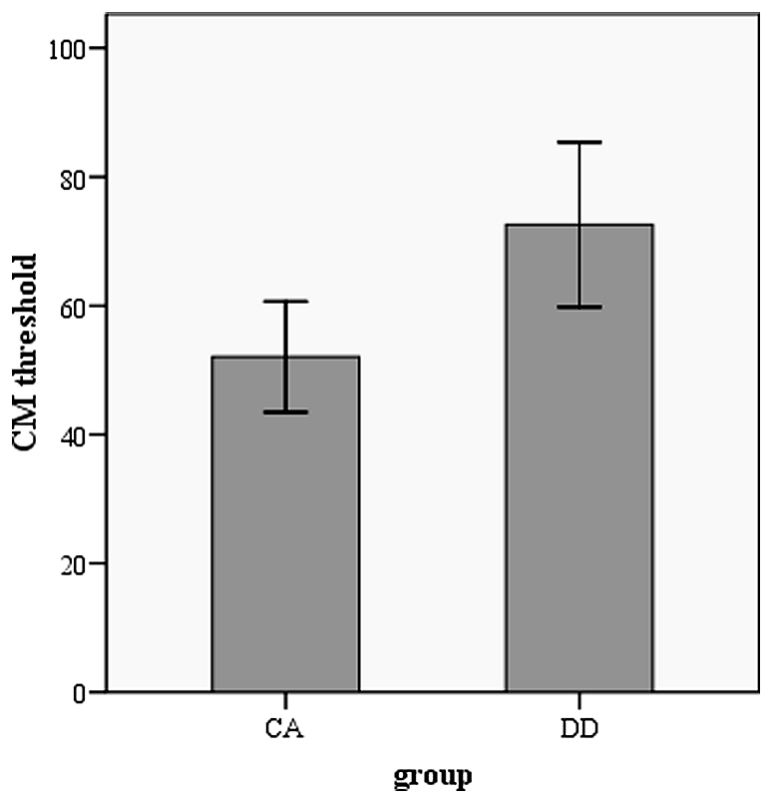
**CM threshold in dyslexia group and age-matched control group**.

**FIGURE 2:**
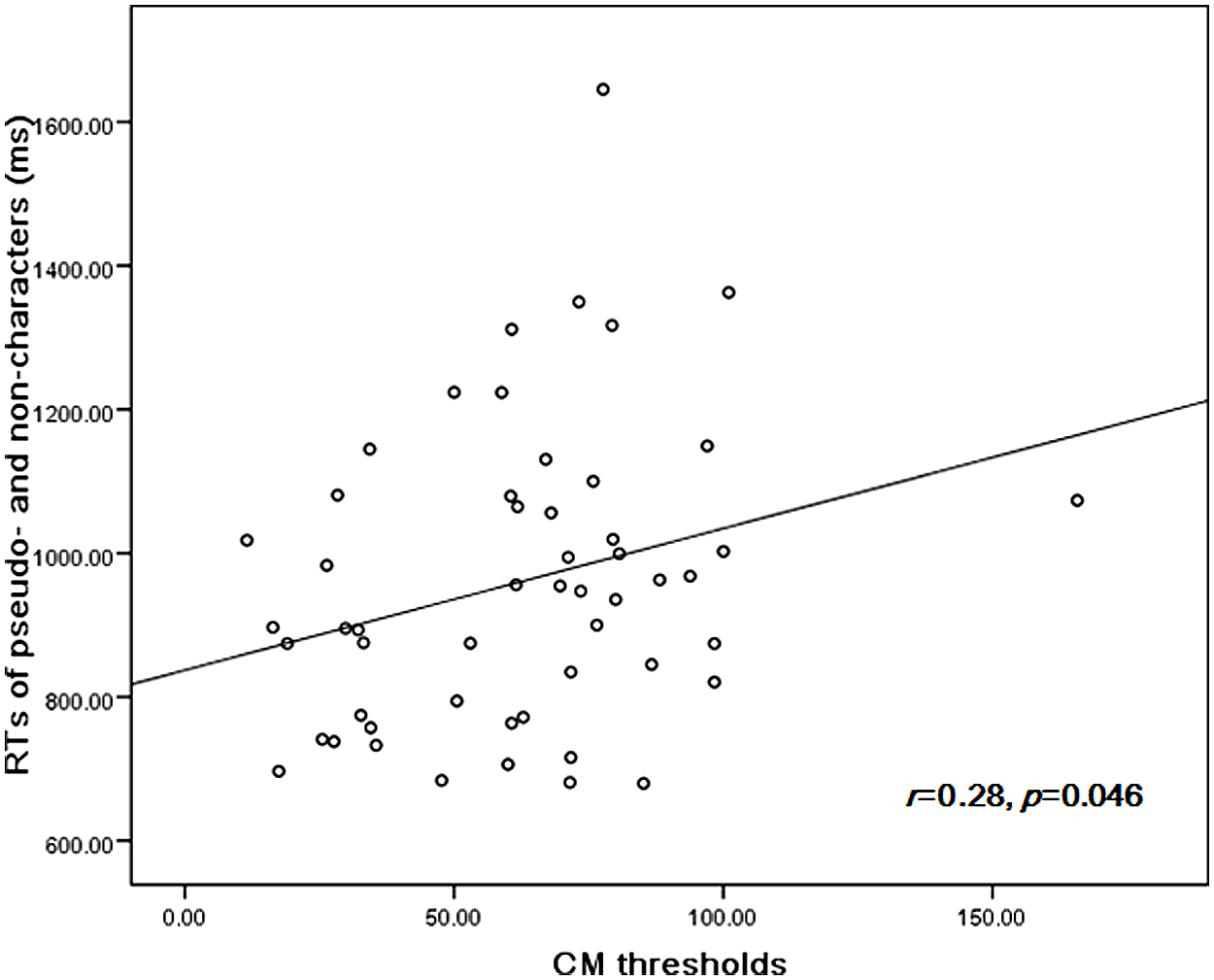
**The correlation between CM thresholds and average RTs of pseudo-characters and non-characters**.

**FIGURE 3:**
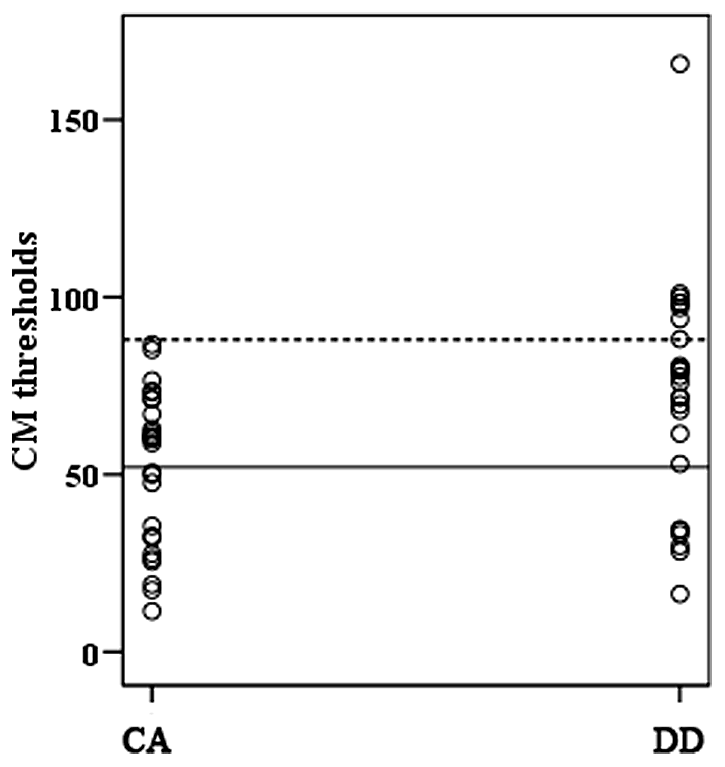
**Individual CM threshold in DD and CA groups**. The solid line indicated the mean of CA group and the dashed line the chosen deviance threshold (1.65 SD above the mean of CA group).

The performance in orthographic awareness test and CM detection task of DD group and age-matched control (CA) group was shown in **Table 2** and **Figure 1**. *t*-test revealed that the CM threshold of DDs was significantly higher than that of CA [*t*(51) = 2.76, *p*< 0.01, *d*= 0.77]. With respect to orthographic awareness, the difference in average accuracy of pseudo-characters and non-characters was not significant between the two groups. The difference in average RTs of pseudo-characters and non-characters was marginally significant [*t*(51) = 1.78, *p*= 0.08, *d*= 0.50], dyslexics responded more slowly than controls. In order to explore the relationship between orthographic processing skills and the performance in CM detection, we made a correlation analysis between average RTs to pseudo-characters and non-characters and CM threshold by merging the data of two groups. As shown in **Figure 2**, the RTs of pseudo-characters and non-characters were significantly correlated with CM threshold (*r*= 0.28, *p*= 0.046). In order to explore whether orthographic awareness influenced the difference in CM thresholds of the two groups, RT in orthographic awareness test was put in a general linear model as a covariate. The results showed that the interaction between RT and group was not significant [*F*(1,49) = 0.18, *p*= 0.68, η^2^ < 0.01]. As shown in **Figure 3**, a deviance analysis was applied to explore the distribution of CM thresholds in DD and CA. There were eight dyslexic children had CM thresholds significantly higher than 1.65 SD above the mean of CA group.

**Table 2:** Performance in CM detection task and orthographic awareness test of dyslexics and age-matched controls.

		DD (*n* = 26)	CA (*n* = 27)	*p* value
CM threshold		72.59 (31.66)	52.07 (21.73)	<0.01
Orthographic	Accuracy	0.78 (0.14)	0.82 (0.12)	0.23
awareness	Reaction time (ms)	1010.48	911.20	0.08

*ACC, accuracy; RT, reaction time.*

## DISCUSSION

The present results showed that Chinese dyslexics had deficits in CM detection and orthographic awareness. Compared with typical children, dyslexic children had higher CM detection thresholds and slower response to pseudo-characters and non-characters. Moreover, the CM thresholds were correlated with average RTs to pseudo-characters and non-characters, suggesting visual M pathway function was closely associated with orthographic processing skills in Chinese-speaking children.

### DEFICITS IN VISUAL M PATHWAY AND ORTHOGRAPHIC PROCESSING

As shown in **Table 2**, dyslexics had significantly higher CM thresholds than age-matched controls. The result was consistent with the findings both in alphabetic languages and Chinese (e.g., Hansen et al., 2001; Conlon et al., 2004; Pellicano and Gibson, 2008; Meng et al., 2011). On account of the insignificant interaction between group and orthographic awareness (as a covariate), the deficits in CM perception of DD were not influenced by the deficient orthographic processing. The deviance analysis revealed that 8 of 26 dyslexic children had thresholds higher than 1.65 SD of control means, suggesting that the percentage of M deficit was relatively small in DD. However, the percentage (about 52%) of CM deficits in Chinese children found by Meng et al. (2011) was higher. The difference of incidence might be related to sampling. The sample size in both studies was too small to investigate the incidence effectively. In the future, larger sample size and more visual M tasks should be adopted to explore the prevalence of M deficits.

Meanwhile, Chinese dyslexics performed more slowly in orthographic awareness test than typical children. In line with the findings of Ho et al. (2004), the results suggested that orthographic processing skills were impaired for Chinese dyslexic children. However, in the present study, the group difference was merely marginally significant in RTs, and not significant in accuracy. One possible reason is that the task (lexical decision task) is easy for children, as their accuracy was about 80%. In addition, a prior study found that orthographic awareness (measured by accuracy) made a unique and significant contribution to Chinese reading for younger children, while the contribution became insignificant after second grade (Wei et al., 2014). Orthographic processing skills might reach a mature level at an early age, which might lead to the less significant differences between dyslexic and typical children at 10 years of age.

### THE RELATIONSHIP BETWEEN M PATHWAY DYSFUNCTION AND DEFICIENT ORTHOGRAPHIC PROCESSING SKILLS

As shown in **Figure 1**, a significant correlation between CM thresholds and RTs in orthographic awareness test was observed in the present study. This finding suggested M pathway function was associated with orthographic processing skills. However, it was just a correlation relationship, and could not reveal causality between M pathway function and orthographic processing skills. As indicated by M deficit theory, it is probable that M deficit causes sluggish orthographic processing. M deficit theory treats M dysfunction as the core cause of dyslexia, which affects a variety of reading skills, including orthographic processing skills (Stein, 2001). M pathway is involved in normal eye movement control, visuospatial attention, visual search, letter position encoding and peripheral vision, which are obviously involved in the development of orthographic skills (Stein, 2001). A longitudinal study, using causal path analysis, found CM detection ability in preschool was related to reading ability in first grade, and the relationship was mediated by orthographic skills (Boets et al., 2008).

There is another possibility that cognitive deficits caused M pathway dysfunction, which was supported by a recent fMRI study. They found the V5/MT activity for dyslexic children was lower than that for age-matched controls, but no different from reading level matched controls. In addition, V5/MT activity for dyslexics increased after phonological-based intervention along with reading gains. The results suggested phonological deficits, by restricting the amount and quality of reading in dyslexics, limited the opportunity for reading to induce changes in the visual M system (although by mechanisms that remained to be determined; Olulade et al., 2013). However, the conclusion was constrained by some confounding factors, such as the small sample size and the visually presented intervention program. Nevertheless, the study of Olulade et al. (2013) provided a possible perspective to explore the causal relationship between cognitive deficits and M pathway dysfunction. In Chinese, will orthographic processing deficits cause the impairment in M pathway? This problem can be investigated in the future by adopting a reading-level matched group and an intervention study.

Butterworth and Kovas (2013) indicated that the same genes might affect multiple traits implicated in diverse cognitive processes. It was possible that the deficits in M pathway and orthographic processing were affected by the same genes. KIAA0319 is a susceptibility gene for dyslexia (Cope et al., 2005; Harold et al., 2006). KIAA0319 is situated within the major histocompatibility complex (MHC) immune control gene complex, which seems to play a particularly important role in the development of M pathway (Stein, 2012). Additionally, FMR1 is also one of dyslexia candidate genes (Poelmans et al., 2011). A study on patients with fragile X syndrome found that the deficient FMR1 gene led to the degeneration of M cells in the lateral geniculate nucleus (LGN; Kogan et al., 2004). Thus, it is reasonable to speculate that deficits of KIAA0319 and FMR1 might give rise to dysfunction in M pathway for children with dyslexia. However, there are no studies to investigate the association between these genes and orthographic awareness. So, it is still unclear whether there is a specific gene to affect both M pathway function and orthographic processing skills. More genetic researches were needed to verify the relationship.

In summary, the current study found Chinese children with DD exhibited deficits both in CM perception and orthographic processing. Moreover, CM thresholds were significantly correlated with RTs of pseudo-characters, suggesting the dysfunction in M pathway was highly associated with impairment in orthographic processing skills.

## Conflict of Interest Statement

The authors declare that the research was conducted in the absence of any commercial or financial relationships that could be construed as a potential conflict of interest.
